# Genistein in the Prevention and Treatment of Periodontitis: A Review

**DOI:** 10.1002/fsn3.70657

**Published:** 2025-08-04

**Authors:** Yujun Lu, Yuye Liang, Zhiyuan Li, Wenxiu Li, Changjun Huang, Yajing Wang, Guangcheng Wang

**Affiliations:** ^1^ The Affiliated Stomatological Hospital of Guizhou Medical University Guizhou Medical University Guiyang China; ^2^ The Stomatology of Guizhou Medical University Guizhou Medical University Guiyang China; ^3^ Guizhou Medical University School of Pharmacy Guizhou Medical University Guiyang China; ^4^ State Key Laboratory of Discovery and Utilization of Functional Components in Traditional Chinese Medicine Engineering Research Center for the Development and Application of Ethnic Medicine and TCM (Ministry of Education) Guiyang China

**Keywords:** anti‐inflammatory, drug delivery system, genistein, periodontitis

## Abstract

Periodontitis is a chronic and progressive inflammatory disease of periodontal tissue whose characteristic pathological changes include the destruction of periodontal soft tissue and the absorption of alveolar bone. Genistein, an isoflavone compound derived from leguminous plants, has pharmacological effects such as anti‐inflammatory, anti‐oxidation, anti‐bacteria, anti‐osteoporosis, anti‐tumor, and estrogen‐like effects. In recent years, genistein has shown broad application potential in the prevention and treatment of periodontitis due to its wide range of biological activities. Therefore, this review presents the mechanism of action of genistein in the prevention and treatment of periodontitis and the different delivery strategies of genistein, aiming to provide ideas for the basic research and clinical application of genistein in taking precautions against and curing the effect of periodontitis.

## Graphical abstract

Genistein, an isoflavone compound with diverse pharmacological activities, shows potential as an adjunctive therapeutic agent for periodontitis due to its anti‐inflammatory, antioxidant, and osteoprotective properties. Genistein not only mitigates the degradation of periodontal soft tissue but also demonstrates a protective effect on alveolar bone by suppressing osteoclast activity and concurrently promoting osteogenic processes. A range of drug delivery systems, including nanoparticles, liposomes, polymer micelles, hydrogels, solid dispersions, and microspheres, have been developed to enhance the bioavailability of genistein, thereby potentially improving its efficacy as an adjunctive therapy for periodontitis.

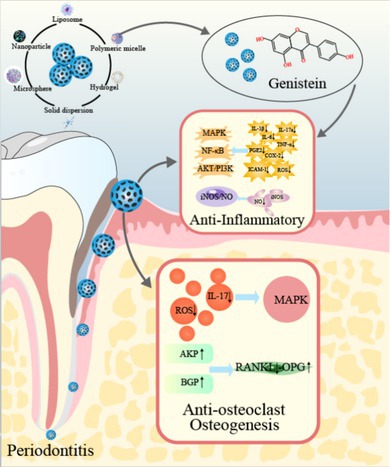

## 1 Introduction

Periodontitis is a chronic inflammatory destructive disease that occurs in periodontal soft and hard tissues. It can cause inflammation of the gums, the formation of periodontal pockets, and the destruction of alveolar bone, which in turn leads to tooth loosening and ultimately tooth loss. The process and progress are determined by the interaction between microorganisms and the host immune defense system (Ramseier et al. 2017). Bacteria and their products in dental plaque are the initiating factors of periodontitis. The antigen components of dental plaque bacteria and the toxic factors such as toxins and enzymes produced by them can directly cause periodontal tissue damage and can also cause host immune response and inflammatory response, indirectly causing periodontal tissue damage (Preshaw et al. 2004).

**FIGURE 1:**
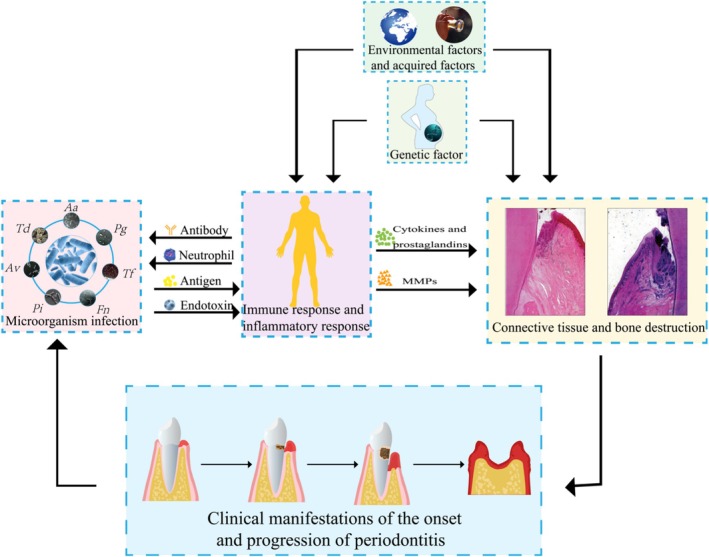
The pathogenic mechanism of periodontitis. The antigen components of dental plaque bacteria (such as *Aggregatibacter actinomycetemcomitans* (*Aa*), *Porphyromonas gingivalis* (*Pg*), *Tannerella forsythia* (*Tf*), *Fusobacterium nucleatum* (*Fn*), *Prevotella intermedia* (*Pi*), *Actinomyces viscosus* (*Av*), *Treponema denticola* (*Td*)) and the toxic factors such as toxins and enzymes produced by them. It can directly cause periodontal tissue damage and can also cause host immune response and inflammatory response (some cytokines, prostaglandins, and matrix metalloproteinases (MMPs) are produced during the reaction), indirectly causing periodontal tissue damage. Whether periodontal infection occurs is determined by three conditions: bacteria, host, and environment. The initial onset and progression of periodontitis exhibit a range of clinical manifestations, encompassing gingival inflammation, the formation of periodontal pockets, alveolar bone resorption, as well as tooth loosening and displacement.

Although dental plaque is ubiquitous within the oral cavity, only a small number of people eventually develop severe periodontitis, indicating that in addition to microbial factors, individual genetic, immune, and environmental factors may also play an important role in the development of periodontitis (Gasner and Schure 2023). Some cytokines, prostaglandins, and matrix metalloproteinases produced during the host immune response can mediate the destruction of periodontal connective tissue and bone tissue. Therefore, periodontitis is classified as a progressive, chronic infectious disease. Its pathological process can lead to persistent inflammation of gingival tissue, accompanied by the formation and deepening of periodontal pockets, which in turn leads to the absorption and destruction of alveolar bone and ultimately leads to the damage of the supporting structure of teeth, and the serious consequences of loosening, displacement, and even shedding of teeth. Figure 1 shows the pathogenic mechanism of periodontitis.

The treatment of periodontitis not only needs to eliminate bacterial infection but also regulates the immune inflammatory response of susceptible hosts, which is one of the key treatments. At present, the treatment of periodontitis often uses mechanical treatment methods such as supragingival scaling and subgingival scaling, but there are certain limitations. For example, it is difficult for instruments to reach certain parts, resulting in poor results. Therefore, drug therapy has become one of the most commonly used adjuvant means of mechanical therapy. Drugs for adjuvant treatment of periodontitis can enhance the effect of basic periodontal treatment and promote the recovery of periodontal health by inhibiting the activity of pathogenic bacteria and regulating the inflammatory response of the host. Common adjuvant drugs include various antibiotics and non‐steroidal drugs, but they are prone to adverse reactions such as drug resistance, gastrointestinal bleeding, cardiovascular and renal effects. And after discontinuation may also appear to accelerate the phenomenon of alveolar bone loss (Preshaw 2018).

Natural plant extracts have shown significant advantages in scientific research due to their high cost‐effectiveness and relatively few side effects and are gradually becoming a hot spot in many research fields. Genistein, as a natural product derived from legumes, is classified as flavonoids. These compounds exhibit rich biological effects and wide biological activities due to their unique chemical structure (Suthar et al. 2001). Genistein also plays a role in periodontal diseases because of its anti‐inflammatory, anti‐osteoporosis, and anti‐bacteria abilities. However, low bioavailability limits the full conversion of in vitro benefits of genistein into clinical applications. Therefore, different drug delivery systems have been developed and applied, such as nanoparticles, liposomes, polymer micelles, hydrogels, solid dispersions, and microspheres (Jaiswal et al. 2019).

This article summarizes the research progress of genistein on periodontitis‐related soft tissue damage and alveolar bone resorption and summarizes the different delivery strategies of genistein, in order to provide a theoretical basis and new ideas for the basic research and clinical application of genistein in the prevention and treatment of periodontitis.

## 2 Brief Introduction of Genistein

### 2.1 Chemistry of Genistein

Genistein, also known as 5,7,4′‐trihydroxyisoflavone, has a molecular weight of 270.24 (C_15_H_10_O_5_). It is an active ingredient widely found in legumes such as soybean, clover, pueraria, sophora, and genistein (Garbiec et al. 2022). Genistein exhibits good solubility in organic solvents such as dimethyl sulfoxide (DMSO) and ethanol, but its solubility in water is extremely low (Dixon 2002). Phytoestrogens encompass a diverse array of subtypes, including isoflavones, coumestans, lignans, chalcones, flavones, and prenylflavonoids. Among these, isoflavones constitute the most prevalent form of phytoestrogens (Ososki and Kennelly 2003).

Genistein, the primary constituent of soybean isoflavones, is recognized as a “phytoestrogen” due to its structural analogy to estrogen. This similarity facilitates its role in maintaining the equilibrium of female hormonal levels (Cooke et al. 2006). Specifically, genistein shares a common phenolic ring base structure with the potent estrogen 17‐β estradiol and exhibits a comparable spatial distance between its 4′‐hydroxyl and 7′‐hydroxyl groups. These structural attributes enable genistein to interact with sex hormone‐binding proteins and estrogen receptors, thereby classifying it as a phytoestrogen (Guelfi et al. 2023). Estrogen receptors are expressed in osteoblasts, osteoclasts, mesenchymal stem cells, osteocytes, the periodontal ligament, and gingival tissues within the periodontium. Estrogen plays a critical role in alveolar bone remodeling by modulating osteoclast activity. Specifically, estrogen therapy reduces osteoclast numbers in alveolar bone, promotes their apoptosis, and inhibits alveolar bone resorption (Faloni et al. 2007). Genistein, a soybean isoflavone and phytoestrogen, may partially mimic estrogenic activity and effectively attenuate the reduction in alveolar bone height associated with estrogen deficiency, thereby exerting a protective effect on periodontal tissues. However, its specific molecular mechanisms remain to be fully elucidated.

**FIGURE 2:**
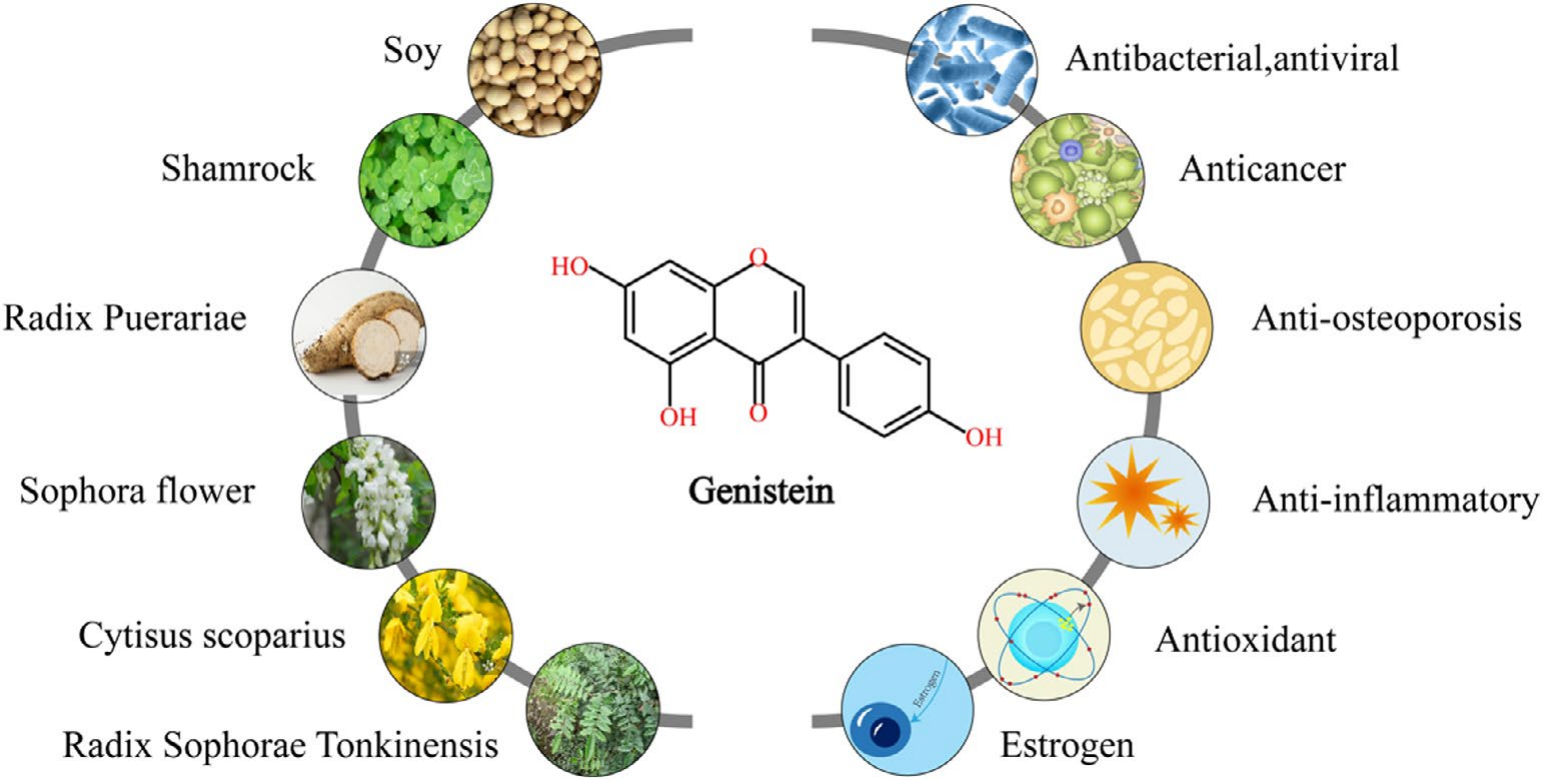
Part of plant sources (left) and pharmacological activities (right) of genistein. Genistein is widely found in soy, shamrock, radix puerariae, sophora flower, *Cytisus scoparius* , radix sophorae tonkinensis, and other leguminous plants. Genistein exhibits a multitude of pharmacological activities, including antibacterial, antiviral, anti‐cancer, anti‐osteoporosis, anti‐inflammatory, antioxidant properties, and estrogenic activity.

As a natural phytoestrogen in the diet, genistein has many effects, such as anti‐inflammatory, anti‐oxidation, anti‐bacteria, anti‐osteoporosis, anti‐tumor, prevention of cardiovascular disease, and improvement of metabolic diseases (Nazari‐Khanamiri and Ghasemnejad‐Berenji 2021). It has many physiological and pharmacological properties, such as regulating insulin, anti‐cancer, and lowering blood lipid in the prevention and treatment of many chronic diseases (Rasheed et al. 2022). At present, genistein‐rich drugs have been widely used in the study of cancer, diabetes, obesity, and other diseases (Sharifi‐Rad et al. 2021). Figure 2 shows some plant sources and pharmacological activities of genistein.

### 2.2 Pharmacokinetics and Safety of Genistein In Vivo

The oral absorption rate of genistein is low and the distribution is limited. Zhou et al. (2008) discussed the pharmacokinetic characteristics of genistein after oral administration of three doses and found that the bioavailability of genistein was relatively low, only 19%–33.5%. Andlauer et al. (2000) found that the absorption rate of genistein in the small intestine was 40.6%, and the average absorption rate was 2.9 nmoL/min g (dry weight of the small intestine). Most of the absorbed genistein existed in the form of glucuronide, which was the main metabolite in the intestinal cavity (13.3%). Only 2.6% of genistein and 2.9% of genistein glucuronide existed in the small intestine. There are few reports on the distribution of genistein in the human body, and the tissue distribution characteristics in rats show significant heterogeneity. Studies have shown that the distribution of genistein in the brain, liver, ovary, prostate, testis, thyroid, and uterus is significantly dose‐dependent, and there are obvious differences in different tissues (Churchwell et al. 2000). Genistein is mainly excreted with urine through the kidney, followed by excretion with bile through the digestive tract.

In terms of safety, genistein is a natural compound derived from plants, which has the potential for endocrine disruption and shows low toxicity and significant pharmacological activity. Ullmann et al. (2005) conducted a first‐stage clinical study on a single oral dose of 30, 60, 150, 300 mg genistein preparation in 40 healthy volunteers to evaluate its safety and tolerance in healthy volunteers. The results of the study demonstrated that genistein was safe and well‐tolerated within the investigated dose range, exhibiting near‐dose linear pharmacokinetic characteristics.

## 3 The Effect of Genistein on Periodontitis

In the pathological process of periodontitis, inflammatory mediators play a pivotal role, causing significant damage to the entire periodontal tissue. These mediators can also trigger soft tissue inflammation and bone damage, which are mediated by both the innate and adaptive immune systems (Ramadan et al. 2020). Studies in vivo and in vitro have found that genistein has the ability to regulate multiple inflammatory and osteogenic signaling pathways. It includes nuclear factor‐κB (NF‐κB), protein serine‐threonine kinase (AKT)/phosphatidylinositol 3‐kinase (PI3K), mitogen‐activated protein kinase (MAPK) and inducible nitric oxide synthase (iNOS)/nitric oxide (NO) and other key pathways. This article summarizes the cytological (Table 1) and zoological studies (Table 2) on the effect of genistein in periodontal disease pathogenesis. Through these regulatory mechanisms, genistein can effectively inhibit the expression of inflammatory mediators such as interleukin‐1β (IL‐1β), tumor necrosis factor‐α (TNF‐α), cyclooxygenase‐2 (COX‐2), and prostaglandin E_2_ (PGE_2_) and show significant anti‐inflammatory effects in periodontal inflammation, thereby inhibiting the further destruction of periodontal tissue.

**TABLE 1:** Cytological studies on the effect of genistein in periodontal disease pathogenesis.

Type of cell	Intervention(s)	Results	References
HGFs	Cells were treated with the genistein (10 μM) for 30 min and later were incubated with LPS (1 μg/mL) for 3 h	Genistein inhibited effect on MAPK activation, IL‐1β, and COX‐2 expression, and PGE_2_ synthesis	Gutiérrez‐Venegas et al. (2007)
HGFs	Cells were treated with genistein (5 μg/mL) for 24 h	Genistein‐induced decrease of PGE_2_ production	Noguchi et al. (1996)
HGFs and RAW 264.7	Cells were treated with 0–70 μM of genistein 1 h before stimulation with 5 μg/mL of LPS for 24 h	Genistein: Attenuated the induction of inflammation‐related molecules (NOS2, COX‐2, TNF‐α, ICAM‐1, MMP‐2, and MMP‐9). Inhibits cellular ROS accumulation	Bhattarai et al. (2017)
RAW 264.7	Cells were treated genistein (50 μM) for 30 min before 24 h incubation with LPS	Genistein: Decreased production of NO and IL‐6. Repressed LPS‐induced iNOS protein expression. Down‐regulated LPS‐induced expression of iNOS and IL‐6 mRNA	Choi et al. (2016)
Neutrophils	Cells were treated with genistein (16.9 μg/mL) for 90 min	Genistein suppresses the NF‐κB activation induced by ROS	Reina et al. (2013)
HPDLCs	Cells were treated with genistein (10 μM) for 0–90 min	Genistein postpones the activation of MAPKs induced by IL‐1β	Luo et al. (2012)
HGFs	Cells stimulated with 10 ng/mL of EGF, were exposed to 20 μM genistein for 15 min prior to a 48 h incubation time	Genistein inhibited EGF‐induced tyrosine phosphorylation of EGF receptors and the activation of ERK and JNK pathways	Smith et al. (2004)

**TABLE 2:** Zoological studies on the effect of genistein in periodontal disease pathogenesis.

Animal model	Intervention(s)	Results	References
Adult male Sprague–Dawley rats, periodontitis were ligature‐induced at the right lower first molars	Genistein (10 mg/kg) was supplied by intraperitoneal injection for 14 days, starting immediately after periodontitis induction	Reduced alveolar bone loss in periodontitis. Genistein prevented changes in trabecular microstructural parameters	Choi et al. (2016)
C57BL/6 male mice, LPS/ligature‐induced periodontitis model	Genistein (20 mg/kg) was supplied by intraperitoneal injection for 3 weeks, starting after periodontitis induction	Reduced alveolar bone loss in periodontitis. Suppresses osteoclast differentiation and inflammation‐related molecule	Bhattarai et al. (2017)
Rats, A rat model of experimental periodontitis with estrogen deficiency was established by silk ligature and inoculation with *Porphyromonas gingivalis*	Genistein was supplied by gavage	Prevented the absorption of alveolar bone. Alleviated tight junction protein expression decreased IL‐17 and ROS levels	Liu et al. (2022)
C57 male mice, periodontitis were ligature‐induced at bilateral maxillary second molar neck	Genistein (20 and 40 mg/kg/day) was supplied by gavage (continuous 4 weeks)	Reduced alveolar bone loss in periodontitis. Reduced the expression levels of TNF‐α, IL‐6, IL‐1β and IL17a in gingival tissues of periodontitis mice	Zhang et al. (2023)
Male Sprague–Dawley rats, periodontitis were ligature‐induced at maxillary first molar neck	Genistein (25 and 75 mg/kg/day) was supplied by gavage (continuous 4 weeks)	Down‐regulated the level of TNF‐α, IL‐1β, IL‐6 in serum. Prevented the absorption of alveolar bone. Decreased number of osteoclasts. Increased expression of OPG and reduced RANKL expression	Dai et al. (2024)
Male Sprague–Dawley rats, periodontitis were ligature‐induced at maxillary first molar neck and high sugar soft food feeding	Genistein (25 and 75 mg/kg/day) was supplied by feeding (continuous 65 day)	Prevented the absorption of alveolar bone. Increased AKP and BGP levels	Yang et al. (2007)

### 3.1 Genistein Inhibits Periodontal Soft Tissue Destruction

Periodontitis is a chronic inflammatory condition whose onset and progression are intimately associated with inflammatory responses. Dental plaque, particularly comprising pathogens, such as *Porphyromonas gingivalis* and actinomycetes, serves as the primary etiological agent. Notably, periodontal pathogens such as *Porphyromonas gingivalis* possess the capability to synthesize toxins by liberating lipopolysaccharide (LPS) components embedded in the outer membrane of Gram‐negative bacterial cell walls. Subsequent to bacterial cell lysis, endotoxin is disseminated into the surrounding tissues, resulting in the degradation of periodontal structures (Marcano et al. 2021). Consequently, LPS, recognized as a key virulence factor in periodontitis, mediates inflammatory processes and tissue degradation by triggering the host immune response, thereby serving as a foundational tool for the development of experimental periodontitis models (Xu et al. 2020).

Inflammation constitutes a fundamental and persistent feature within the pathological cascade of periodontal disease, encompassing its entire spectrum from initiation to progression (Loos and Van Dyke 2020). Genistein inhibits the production of PGE_2_ and IL‐1β in LPS‐stimulated human gingival fibroblasts (HGF) (Noguchi et al. 1996). Studies have also shown that genistein significantly reduced the expression levels of TNF‐α, IL‐6, IL‐1β, and IL‐17a in the gingival tissue of periodontitis mice and reduced the damage of inflammatory factors to periodontal tissue (Zhang et al. 2023).

Nitric oxide (NO) is a free radical gas that serves as a biomarker of inflammation. In physiological states of health, a delicate equilibrium exists between free radicals and their scavengers. However, when the generation of free radicals escalates, this balance shifts towards an unhealthy pro‐oxidant milieu, resulting in significant cellular damage and contributing to the progressive inflammatory diseases of periodontal tissues (Parwani and Parwani 2015). Gutiérrez‐Venegas et al. (2007) found that genistein could inhibit the synthesis of NO induced by LPS in human gingival fibroblasts, hinder the activity of the MAPK signaling pathway, inhibit the expression of COX‐2 and IL‐1β and the synthesis of PGE_2_, and had no effect on cell viability and genetic material integrity. They also found that genistein could significantly inhibit the accumulation of iNOS‐derived NO and IL‐6 in RAW 264.7 cells induced by *Prevotella intermedia* LPS and reduce their mRNA expression, indicating that genistein inhibited these inflammatory mediators at the level of gene transcription and translation (Choi et al. 2016).

In addition, the effect of genistein on LPS‐induced inflammation and oxidative stress in RAW 264.7 macrophages and human gingival fibroblasts was also studied. The results showed that genistein could dose‐dependently inhibit LPS‐stimulated RAW 264.7 macrophages and human gingival fibroblasts. Oxidative stress (such as mitochondrial damage and cellular reactive oxygen species (ROS) accumulation) and the expression of inflammation‐related molecules (such as COX‐2 and ICAM‐1) confirmed that genistein inhibited LPS‐mediated periodontal tissue destruction (Bhattarai et al. 2017). Reina et al. (2013) found that genistein can significantly inhibit the production of ROS in neutrophils, reflecting its potential antioxidant and anti‐inflammatory effects. However, the regulatory relationship between genistein and destructive ROS production under conditions such as periodontal disease needs further study.

Luo et al. (2012) confirmed the expression of intracellular transmembrane G protein‐coupled estrogen receptor GPR30 in human periodontal ligament cells. GPR30 is a new target for steroid hormones to regulate MAPK activity in human periodontal ligament cells. Inflammatory cytokine IL‐1β can induce the expression of GPR30 and activate MAPK, NF‐κB and PI3K signaling pathways in these cells. Genistein can delay the activation of MAPKs in human periodontal ligament cells through GPR30, which can provide a new therapeutic idea for the intervention therapy of periodontitis. Moreover, Smith et al. (2004) found that genistein treatment of human gingival fibroblasts effectively inhibited epidermal growth factor (EGF)‐induced tyrosine phosphorylation of EGF receptor and activation of extracellular signal‐regulated kinase (ERK) and c‐Jun N‐terminal kinase (JNK) pathways, suggesting that genistein can be used as a potential preventive/therapeutic agent to control periodontal inflammation.

### 3.2 Genistein Inhibits Alveolar Bone Destruction

Alveolar bone loss signifies the advancement of periodontitis (Zhu et al. 2022). Addressing the challenges in periodontitis treatment involves preventing alveolar bone resorption and achieving a measurable degree of alveolar bone regeneration, which remains formidable tasks. Osteoclasts, the primary cells responsible for bone resorption, differentiate from monocyte/macrophage precursors under the regulation of key cytokines such as macrophage colony‐stimulating factor, RANK ligand, and osteoprotegerin. In the periodontal inflammatory microenvironment, specific cytokines play a pivotal role in bone resorption by modulating osteoclast formation, thereby contributing to bone destruction in periodontitis. TNF‐α, IL‐1, and PGE_2_ also stimulate osteoclast activity, especially in inflammatory conditions characterized by osteolysis, such as those observed in periodontitis (Hienz et al. 2015).

Thent et al. (2018) suggested that phytoestrogens can regulate vital biomarkers of bone mineralization, so they can be used as an alternative bone protectant. Genistein, a phytoestrogen, exhibits a significant anti‐osteoporotic effect. According to research, Genistein can not only effectively inhibit inflammatory bone resorption, but it also has the ability to promote bone formation and has a strong bone protection effect (Li et al. 2013). This finding may imply that genistein plays a crucial role in suppressing alveolar bone resorption associated with periodontitis. Although estrogen can prevent alveolar bone loss and maintain bone mass in elderly or postmenopausal women with periodontitis, its use may also increase the risk of breast cancer and endometrial cancer among drug users (Giuca et al. 2009). Therefore, it is particularly important to use natural, safe, and effective substances as alternative therapies to reduce the side effects of traditional treatments.

#### 3.2.1 Genistein Inhibits Osteoclastogenesis Process

Receptor activator of nuclear factor‐κB ligand (RANKL) indeed plays a crucial role in osteoclast formation. It binds to receptor activator of nuclear factor‐κB (RANK) on osteoclasts to increase the activity of osteoclasts (Elango et al. 2021). Osteoclastogenesis is regulated by osteoblasts through the production of RANKL and osteoprotegerin (OPG). OPG is a decoy receptor for RANKL and inhibits osteoclast differentiation and function by blocking the interaction between RANKL and RANK in osteoclasts (Khosla 2001). The ratio of RANKL/OPG increased in periodontal tissues of patients with periodontitis, suggesting that the balance between osteoclasts and osteoblasts was disturbed (Ateeq et al. 2022).

Studies have found that genistein can inhibit the differentiation of mononuclear macrophages into osteoclasts by regulating the RANKL/OPG signaling pathway, reduce osteoclast activity, reduce bone resorption, and thus reduce alveolar bone destruction caused by periodontitis. Bhattarai et al. (2017) showed that intraperitoneal injection of genistein (20 mg/kg) every day for 3 consecutive weeks could inhibit LPS‐mediated alveolar bone loss and periodontal tissue degradation. The protective effect on alveolar bone is achieved by regulating RANKL. Similarly, through micro‐CT analysis, Choi et al. (2016) found that genistein reduced the loss of alveolar bone height and bone volume fraction in rat periodontitis induced by ligation and also prevented changes in trabecular microstructure parameters (trabecular thickness, trabecular separation, bone mineral density, and structural model index), suggesting that genistein may potentially be utilized in the future for the treatment of human periodontitis.

Zhang et al. (2023) found that administration of genistein (20 and 40 mg/kg) can reduce alveolar bone resorption in periodontitis mice induced by silk ligation, which may be related to its bone protection. In addition, Dai et al. (2024) confirmed that genistein reduced osteoclast formation. In the study of mechanism, it was found that genistein could inhibit the expression of RANKL and increase the expression of OPG in periodontal tissue of rats with periodontitis. It could also reduce the levels of Slit homolog 2 (Slit2) and P38 MAPK phosphorylated protein in rats with periodontitis, so as to draw a conclusion. Genistein can reduce periodontal tissue inflammation and alveolar bone resorption in periodontitis rats. The mechanism may be related to the inhibition of Slit2 / P38 MAPK signaling pathway, regulation of RANKL/OPG expression, and inhibition of osteoclast formation.

Interleukin‐17 (IL‐17) is an important cytokine secreted by Th17, which has the function of stimulating human periodontal ligament cells to express RANKL and promote osteoclast differentiation (Ikeuchi and Moutsopoulos 2022). Estrogen deficiency leads to a downregulation of genes encoding tight junction proteins in the gingiva, an elevation of IL‐17 levels, and an acceleration of alveolar bone resorption. Studies have found that genistein treatment can alleviate the gene levels of IL‐17 in ovariectomized experimental periodontitis rats, increase the expression of tight junction proteins, and prevent the absorption of alveolar bone (Liu et al. 2022).

#### 3.2.2 Genistein Promotes Osteogenic Process

As a safe and effective “phytoestrogen,” genistein can promote osteoblast proliferation, differentiation, and mineralized bone formation and inhibit osteoclast bone resorption. There are many molecular mechanisms of anti‐osteoporosis. It can selectively bind to estrogen receptor (ER) in vivo and play a role in the prevention and treatment of osteoporosis. The mechanism may be to promote the proliferation, differentiation and mineralization of osteoblasts through PPARγ, BMP/Smads/Runx2, MAPK/NF‐κB/AP‐1 and other signaling pathways. At the same time, it can inhibit osteoclast bone resorption through NF‐κB pathway and intracellular calcium signaling pathway (Zheng et al. 2016). Furthermore, genistein has an anabolic effect on osteoblast MC3T3‐E1 in vitro (Sugimoto and Yamaguchi 2000). Furthermore, numerous studies have demonstrated that genistein possesses the ability to enhance bone regeneration and elevate bone density (Chan et al. 2020).

**FIGURE 3:**
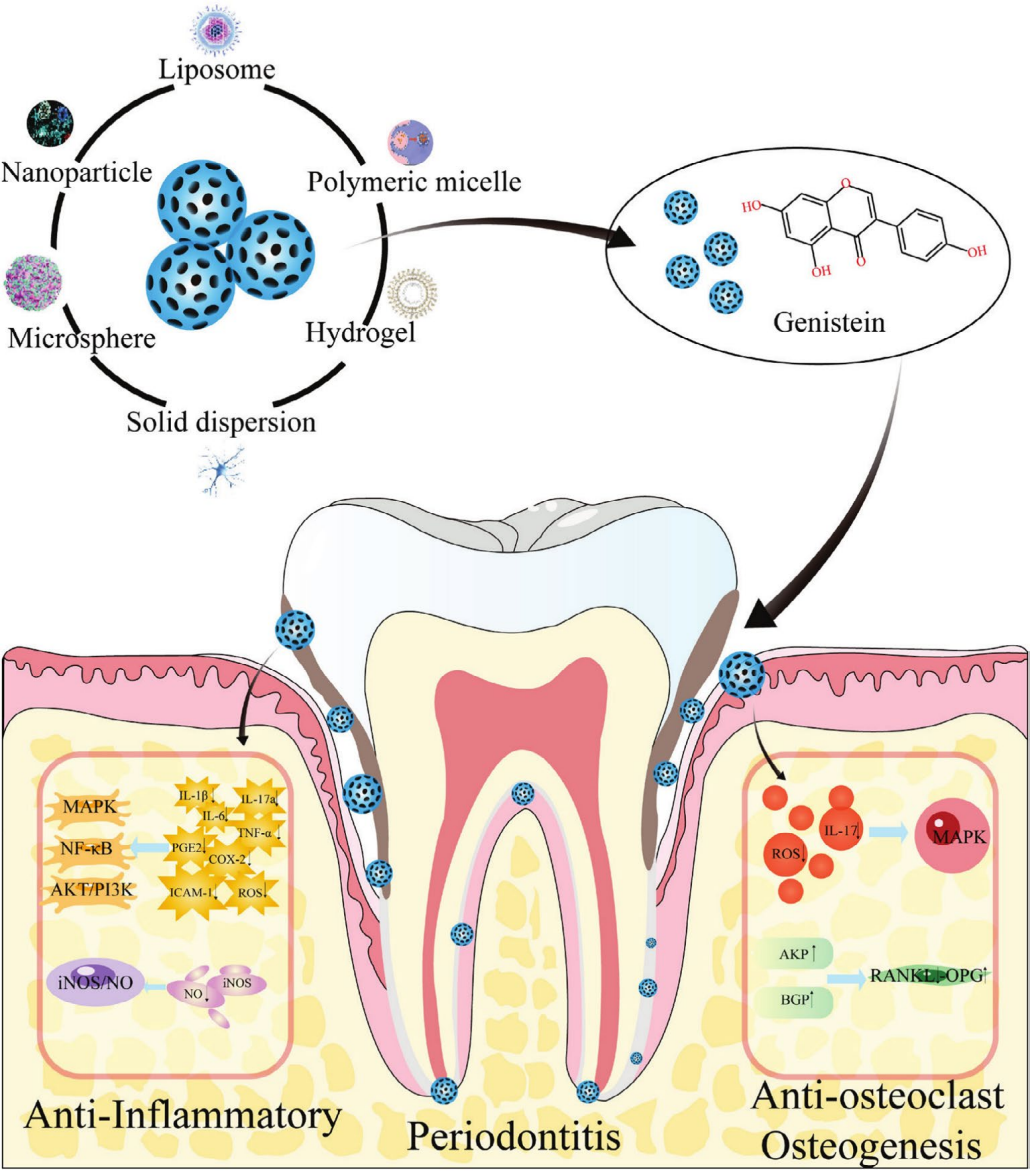
Overview of the mechanism of genistein in the treatment of periodontitis. Novel drug delivery systems for genistein, such as nanoparticles, liposomes, polymer micelles, hydrogels, solid dispersions, and microspheres, exhibit exceptional solubility, targeting, and sustained release, overcoming its poor water solubility and enabling effective periodontitis treatment. In periodontitis, genistein potently suppresses inflammatory mediators (IL‐1β, TNF‐α, etc.), exhibiting anti‐inflammatory activity. It also modulates bone metabolism by inhibiting RANKL and enhancing OPG, AKP, and BGP expression, contributing to bone protection. Genistein regulates multiple inflammatory and osteogenic signaling pathways such as MAPK, NF‐κB, AKT/PI3K, RANKL/OPG, and iNOS/NO. ↑, Increase; ↓, Decrease; AKP, Alkaline phosphatase; AKT/PI3K, Proteinserine‐threonine kinase/phos‐phatidylinositol3‐kinase; BGP, Bone gla protein; COX‐2, Cyclooxygenase‐2; ICAM‐1, Intercellular adhesion molecule‐1; IL‐17a, Interleukin‐17a; IL‐1β, Interleukin‐1β; IL‐6, Interleukin‐6; iNOS/NO, Inducible nitric oxide synthase/nitric oxide; MAPK, Mitogen‐activated protein kinase; NF‐κB, Nuclear factor‐κB; OPG, Osteoprotegerin; PGE_2_, Prostaglandin E_2_; RANK, Receptor activator of nuclear factor‐κB; RANKL, Receptor activator of nuclear factor‐κB ligand; ROS, Reactive oxygen species; TNF‐α, Tumor necrosis factor‐α.

As periodontitis progressively worsens, it leads to irreversible damage to the alveolar bone. This pathological change is highlighted by the breakdown of the original balance between osteoclast‐driven bone decomposition (bone resorption) and osteoblast‐driven bone synthesis (bone formation) (Hienz et al. 2015). Guo et al. established a rat model of experimental periodontitis with osteoporosis and found that genistein effectively improved the changes in biochemical indexes of bone metabolism caused by estrogen deficiency. The levels of alkaline phosphatase (AKP) and bone gla protein (BGP) were significantly increased, indicating that genistein contributes to bone formation and the maintenance of normal bone calcification and effectively prevents alveolar bone resorption caused by estrogen deficiency in rats with periodontal disease, which can play a role in alternative estrogen therapy (Yang et al. 2007). Figure 3 is an overview of the mechanism of genistein in the treatment of periodontitis.

### 3.3 Genistein Inhibits Bacterial Process

Periodontal disease is characterized by a mixed bacterial infection that results in inflammatory destruction of the periodontal tissues that support and surround the teeth. The periodontal microbiota comprises a complex dental plaque biofilm ecosystem, where pathogenic bacteria produce virulence factors that allow them to evade host defenses and elicit a detrimental immune response in the host tissues. Periodontal disease arises due to an imbalance or disruption in the normal homeostasis among plaque bacteria, the host immune system, and the environmental conditions present in a healthy state (Harvey 2017). More than 700 different microorganisms have been isolated from the human oral cavity, including aerobes, facultative anaerobic bacteria, and obligate anaerobic bacteria, as well as other microorganisms such as spirochetes, fungi, archaea, mycoplasma, viruses, and protozoa (Zhang et al. 2018). Currently, the antibacterial agents frequently employed in the treatment of periodontitis encompass nitroimidazoles, tetracyclines, penicillins, and macrolides; however, these agents are prone to inducing the emergence of drug‐resistant strains and may elicit adverse effects, including gastrointestinal disturbances and systemic allergic reactions (Soares et al. 2012).

Genistein exhibits antibacterial properties, demonstrating its potential as an antimicrobial agent. A study suggested that *Staphylococcus aureus* , a medically important pathogen, was found at a high prevalence in non‐smoking individuals with aggressive periodontitis. Moreover, a diagnostic utility distinguishing between aggressive and chronic periodontitis sites was found for *P. intermedia* , *S. aureus* , and *S. mutans* (Fritschi et al. 2008). In addition, *S. aureus* is an early‐colonizing bacterium of titanium surfaces (Fürst et al. 2007), and a pathogen in periimplantitis (Leonhardt et al. 1999). A standardized in vitro experiment was conducted to assess the direct impact of genistein on bacterial growth. In bacterial colony‐forming unit (CFU) assays, notable decreases in CFU counts were observed for *Staphylococcus aureus* and *Bacillus anthracis* when cultivated in the presence of 100 μM genistein. Additionally, the incorporation of genistein alongside probiotics has the potential to enhance the efficacy of existing antimicrobial treatments utilized in infection management (Hong et al. 2006). In another study by Guo (2023), the impact of genistein on MRSA (methicillin‐resistant *S. aureus* )‐induced osteomyelitis was examined in male Wistar rats. The results indicated a decrease in pro‐inflammatory cytokines, specifically IL‐6 and TNF‐α, as well as a reduction in bacterial growth.

*Helicobacter pylori* (*Hp*) serves as a pathogen for chronic gastritis and gastric ulcer, and recent research has demonstrated that dental plaque functions as a reservoir for this bacterium. Specifically, *Hp* can be identified within periodontal pockets, with a notably higher detection rate observed in cases of gingival bleeding compared to non‐bleeding instances (Maurotto et al. 2024). One study showed that the risk for positive in the periodontitis test was higher among subjects with *Hp* infection as compared with those without, while post‐eradicated status tended to reduce that risk (Adachi et al. 2019). A study has demonstrated that genistein possesses potent antibacterial activity in vitro. Exposure to genistein exhibited an inhibitory effect on all staphylococcal strains tested. Furthermore, the growth of *Hp* was clearly inhibited by genistein (Verdrengh et al. 2004). Research findings have demonstrated that genistein exerts gastroprotective effects in rats with *Hp‐induced* gastropathy by means of reducing the levels of pro‐inflammatory mediators, downregulating the expression of the nuclear receptor NF‐κB, and mitigating gastric mucosal apoptosis (Siriviriyakul et al. 2020).

Up to now, there has been a lot of evidence to fully support *Aggregatibacter actinomycetemcomitans* , *Porphyromonas gingivalis* , and *Tannerella forsythia* as the key pathogenic bacteria of periodontal disease (Ursu et al. 2022). These bacterial strains occupy a central position in the development of periodontal disease, especially concerning the degradation of periodontal tissues. However, there is still a lack of research on the interaction between genistein and the above important pathogenic bacteria related to periodontitis, and there are few reports in the relevant scientific literature. Given that genistein is a natural compound with potential antibacterial activity, its inhibitory effect on certain bacteria in vitro experiments suggests that the antibacterial properties of genistein are worthy of further exploration, especially in the intervention strategy for the pathogenesis of periodontitis. Therefore, future research directions can focus on elucidating the specific mechanism of action between genistein and periodontitis pathogens and evaluating its potential as an adjuvant treatment for periodontal disease, thus paving the way for innovative approaches in the prevention and management of periodontal disease.

### 3.4 Clinical Study

A clinical study was conducted on 3956 students (aged 18–22 years) to collect information on dietary factors using a validated dietary history questionnaire. Logistic regression analysis was employed to ascertain the odds ratio and corresponding confidence interval associated with periodontal disease. Smoking, brushing frequency, place of residence, and body mass index were adjusted. The results showed that the intake of foods containing genistein may reduce the possibility of periodontal disease (Tanaka et al. 2008). Currently, a substantial absence of extensive clinical investigations into genistein's application for periodontitis treatment persists, necessitating additional confirmatory trials to advance its clinical adoption.

In conclusion, genistein exerts therapeutic effects in periodontitis through three key mechanisms: (1) suppressing periodontal soft tissue inflammation, (2) inhibiting osteoclast activity while promoting osteoblast function to reduce alveolar bone resorption, and (3) inhibiting the growth of specific pathogenic bacteria implicated in the disease. Although genistein exhibits a notable therapeutic effect in the treatment of periodontitis, current research on its mechanisms for inhibiting alveolar bone resorption and its antibacterial properties in periodontitis remain limited, necessitating further investigation.

## 4 Common Delivery Systems of Genistein

In recent years, genistein has attracted much attention in the field of oral medicine as a potential natural drug. Improving the solubility and bioavailability of genistein has important guiding significance for further exploring other related parameters of its pharmacokinetics, which not only helps us to understand the absorption, distribution, metabolism, and excretion process of genistein in vivo more comprehensively, but also provides a scientific basis for optimizing the design of pharmaceutical preparations, improving drug efficacy, and reducing the risk of adverse reactions in the adjuvant treatment of periodontitis.

With the aim of enhancing the bioavailability of genistein, a variety of strategies have been implemented, among which nanoparticles, liposomes, polymer micelles, hydrogels, solid dispersions, and microspheres are the most widely used. We hope that by optimizing the drug delivery system of genistein, its solubility can be improved and its pharmacological activity may be enhanced, thus showing more excellent efficacy in the protection and treatment of periodontal soft and hard tissues. Table 3 is the common transfer system of genistein and the comparison of the characteristics between different transfer systems.

**TABLE 3:** Comparison of characteristics of different delivery systems of genistein.

Transmission system	Size	Advantages	Disadvantages	Dosage form	Bioavailability	References
Nanoparticle	10–1000 nm	Good biodegradability, compatibility, and safety. Drug targeting and controlled release	Some reaction steps are complicated. High surface activity is easy to adsorb and agglomerate. Stability is susceptible to environmental factors	Slow release preparation Solid dosage forms (capsules, granules) Semi‐solid dosage form (gel)	The relative bioavailability of genistein from the nanoparticles compared with the reference suspension was 241.8%	Gavin et al. (2015), Bansal et al. (2024), Tang et al. (2011)
Liposome	25–5000 nm	Simple preparation and higher safety. Improve drug solubility	Lack of physical and chemical stability Easy fusion and leakage of drugs	Slow release preparation Liquid dosage forms (gargle, oral, rinse)	*C*_max_ of genistein liposome (1.194 ± 0.054 μg/mL) was significantly higher (*p* < 0.05) than that of the genistein suspension (0.231 ± 0.053 μg/mL)	Almeida et al. (2020), Komeil et al. (2021)
Polymeric micelle	20–200 nm	Long systemic circulation time. Increase intracellular uptake. High encapsulation efficiency	Part of the cost is high, the process is more complex. Initial drug burst release. Easy to adsorb toxic chemicals	Slow release preparation Liquid dosage forms (gargle, oral, rinse)	Genistein‐loaded mixed micelles system showed a 2.42‐fold increase in relative oral bioavailability compared with free genistein	Ghezzi et al. (2021), Shen et al. (2018)
Hydrogel	/	Easy to achieve local administration of drugs	Poor biodegradability in part. Some potential toxic effects	Slow release preparation Semi‐solid dosage form (gel)	Genistein was incorporated into transferosomal hydrogel to improve its bioavailability	Zagórska‐Dziok et al. (2021), Motawea et al. (2024)
Solid dispersion	/	Reduce lipid accumulation. Increase dissolution rate. High oral bioavailability. Method simple and efficient	Low stability. Need to take frequently	1 Solid dosage forms (capsules, granules) 3 Spray formulation	The *C*_max_ and AUC_0–24_ of genistein solid dispersion were increased 6.86‐ and 2.06‐fold to that of pure genistein	Qiu et al. (2024)
Microsphere	1~500 μm	Long time drug controlled release. Biodegradability and high drug loading capacity.	Poor stability. Complex preparation	1 Slow release preparation 3 Solid dosage forms (capsules, granules)	Significant differences in AUC were observed between microsphere genistein and ordinary genistein	Wu and Li (2002a), Rahmani et al. (2023)

### 4.1 Nanoparticle

Nanoparticles are solid‐state drug‐loaded colloidal particles prepared by nanotechnology. The particle size ranges from 10 to 1000 nm, and the drug is encapsulated by dissolution, adsorption, or encapsulation. As a nano‐controlled release system, nanoparticles can significantly improve the targeting, bioavailability, and therapeutic effect of drugs and significantly enhance the water solubility, storage stability, and release controllability of drugs. This technology not only reduces the dose of administration, reduces the side effects of drugs, but also opens up an important way for the development and utilization of drugs with poor water solubility (Liu et al. 2023; Mitchell et al. 2021).

Tang et al. (2011) prepared genistein nanoparticles using nanoprecipitation technology. In contrast to genistein suspensions, the nanoparticle formulations effectively encapsulate the drug within suitable carrier materials to create novel nanoparticle preparations. These preparations substantially optimize the in vitro dissolution characteristics of genistein, leading to a significant enhancement in its bioavailability. With the aim of achieving targeted drug delivery, Patra et al. (2022) employed nanoprecipitation technology to synthesize nanoparticles encapsulated with genistein. This formulation enhanced the extent to which genistein was internalized by cells, prolonged the duration of drug release, and ultimately resulted in an improvement in its anti‐cancer effectiveness. Alam and Akhlaq (2016) formulated nanoparticles encapsulating genistein with the aim of enhancing its bioavailability. The nanoparticles obtained exhibited an average particle size of 123 nm, exhibiting superior drug release characteristics in comparison to conventional dosage forms, while also possessing a high encapsulation efficiency.

A different research team developed MIL‐100(Fe) metal–organic framework nanoparticles via a straightforward impregnation technique, aiming to enhance the oral bioavailability of genistein. The resultant formulation exhibited sustained release of genistein over a 3‐day period, coupled with an improvement in bioavailability, thereby establishing itself as an effective drug delivery strategy (Botet‐Carreras et al. 2021). Zhang et al. (2015) fabricated genistein‐loaded nanoparticles specifically designed for enhanced delivery to target cells. The findings indicated that these nanoparticles exhibited heightened efficacy in inhibiting tumor growth. The results imply that nanoparticles represent promising formulations for the effective delivery of genistein.

Given the limited application potential of genistein in the treatment of periodontal disease due to its poor water solubility and other unfavorable physical properties, it is proposed to utilize solid lipid nanoparticle technology for its encapsulation. The objective is to increase the drug concentration in both plasma and cells, thereby enhancing the bioavailability of genistein. Additionally, this technology is expected to improve the aqueous solution stability and targeting abilities of genistein, ultimately leading to an amplification of its therapeutic efficacy.

### 4.2 Liposome

Liposomes are a special class of vesicles formed by phospholipids. They have a structure similar to that of ordinary vesicles, which can effectively disperse fat‐soluble drugs in aqueous media. When the fat‐soluble drug is encapsulated by liposomes, it can exhibit excellent characteristics such as targeting, sustained release, and stability and has low toxicity. It is a new and advanced drug delivery system (Large et al. 2021). The encapsulation of genistein within liposomes can appreciably enhance an array of its physical and chemical properties, such as solubility, stability, and bioavailability, thereby optimizing its therapeutic potential and enhancing its effectiveness in various biomedical applications.

Research has proposed a novel microgel oral delivery system, loaded with liposome nanoparticles containing a natural anti‐inflammatory compound genistein into alginate microgels, thereby achieving the targeted release of genistein in the colonic region and ameliorating ulcerative colitis symptoms (Yan et al. 2023). Furthermore, Phan et al. (2013) formulated genistein‐encapsulated liposomes and demonstrated their efficacy in enhancing the solubilization and stability of genistein, as well as prolonging its release profile. A research team formulated azolectin‐encapsulated genistein liposomes to explore the interplay between genistein and azolectin. These liposomes exhibited heightened in vitro activities, thereby amplifying their antitumoral effects (Lopes de Azambuja et al. 2015). In a combinatorial or multifunctional approach aimed at treating prostate cancer, a liposomal formulation was developed that incorporated genistein along with celecoxib (Tian et al. 2019) and plumbagin (Tian et al. 2021). Studies conducted on this formulation revealed a significant enhancement in its efficacy for addressing prostate cancer. Additionally, in a separate study, phytosomes loaded with genistein were engineered to improve the solubility of the compound. In vivo experiments further demonstrated that these phytosomes exhibited an elevated anti‐tumor effect (Komeil et al. 2021).

In the realm of periodontitis treatment strategies, consideration is given to employing bovine serum albumin for the construction of a long‐circulating drug delivery system for genistein liposomes. This approach leverages their multiple advantages, including reduced phagocytosis, sustained release properties, improved stability, and non‐toxic nature. Furthermore, by incorporating targeted liposome preparation technology, it is expected that the therapeutic efficacy of genistein in treating periodontitis will be significantly improved, ultimately resulting in more targeted and effective treatment outcomes.

### 4.3 Polymeric Micelle

Polymeric micelles represent a class of self‐assembled nanoparticles characterized by a hydrophobic core encapsulated by a hydrophilic shell. As a widely researched nano‐drug delivery system, targeted polymeric micelles have demonstrated substantial value in clinical applications owing to their exceptional tissue permeability, which is often attributed to their unique structural features and ability to facilitate the delivery of therapeutic agents to specific sites within the body (Hwang et al. 2020). The encapsulation of genistein within polymeric micelles can enhance diverse physicochemical properties of the drug, ultimately optimizing its therapeutic efficacy.

Research studies have employed micelle‐like nanoparticles as a carrier system for encapsulating genistein, with the aim of enhancing the therapeutic efficacy in the treatment of ocular neovascularization (Kim et al. 2012). Similarly, Shen et al. (2018) designed mixed micelles encapsulating genistein to overcome its solubility challenges and augment its bioavailability. Their findings revealed a high degree of entrapment efficiency and drug loading capacity. Cheng et al. (2020) formulated micelles containing genistein by utilizing a modified emulsion‐evaporation method. In vitro studies conducted on this formulation revealed enhanced solubility and bioavailability of genistein.

In a separate study, Kwon et al. (2007) formulated micelles encapsulating genistein using the solid dispersion technique. Their findings revealed an improved drug loading efficiency and an enhanced release profile for genistein, ultimately resulting in an increased bioavailability. Hou et al. (2021) designed glycyrrhizin‐based micelles as a nanocarrier system for genistein, with the objective of augmenting its therapeutic efficacy. In vivo studies have demonstrated the exceptional tolerability and the improved corneal penetration exhibited by the genistein‐loaded micelles. Thus, micelles have been identified as an efficacious strategy for enhancing the therapeutic availability of genistein.

In the treatment of periodontitis, an acid‐responsive genistein polymer conjugated with 1,4,4′‐trimethylenedipiperidine and polyethylene glycol acrylate can be designed. Upon incorporating genistein into its hydrophobic backbone, this polymer self‐assembles into micelles in aqueous conditions due to its amphiphilic nature. Importantly, it rapidly releases genistein under acidic conditions. This design addresses the issue of genistein's poor water solubility and possesses a pathological stimulation response ability for controlled release, thereby offering a promising new strategy for the treatment of periodontitis.

### 4.4 Hydrogel

Hydrogel is a semi‐solid gel with water as the dispersion medium. It is usually a hydrophilic three‐dimensional network structure. It is a crosslinked polymer network that is swollen by water, and the content is usually higher than 90% (Wu et al. 2021). Hydrogel exhibits a wide range of potential applications within the medical field, including but not limited to tissue engineering, wound healing, controlled drug delivery systems, and as biodegradable scaffolds for cell culture and implantation. Encapsulating genistein within hydrogels can optimize its therapeutic effect by safeguarding it from degradation and facilitating its controlled release in vivo. This encapsulation technique enhances the stability and bioavailability of genistein, thereby maximizing its therapeutic potential.

Yang et al. (2021) used genistein encapsulated in hydrogels to alleviate the symptoms of vaginal atrophy and confirmed that the combination of genistein and hydrogels can achieve sustained release of genistein. It has the advantages of strong mucosal affinity and prolonged local retention time and is suitable for transdermal and mucosal administration. In a study, a local hydrogel containing genistein‐loaded nanoemulsion was obtained by spontaneous emulsification. Nanoemulsion‐based hydrogels incorporating genistein could be regarded as a promising delivery system for isoflavones to the skin (de Vargas et al. 2012). In addition, Zagórska‐Dziok et al. (2021) synthesized a new poly (chitosan‐ester‐ether‐carbamate) hydrogel, which is noncytotoxic to skin cells (keratinocytes and fibroblasts) and has a relatively highly controlled release curve. It is considered to be an effective dermatological and/or cosmetic tool. In another study, Mahajan et al. (2023) formulated a keratin‐genistein‐based hydrogel for wound healing and showed anti‐inflammatory properties, suggesting its potential as a promising therapeutic agent for managing wound repair.

The development of a controlled release genistein hydrogel offers several advantages in the treatment of periodontitis. The sustained release characteristic of the hydrogel preparation allows for long‐term antibacterial activity in vitro. Additionally, its bioadhesive property and injectability enhance the topical delivery of natural plant extracts, particularly those containing genistein, into periodontal pockets. This formulation facilitates the insertion of the hydrogel into the periodontal pocket during experimental procedures, ensuring precise placement. Additionally, the hydrogel's controlled release mechanism allows for sustained drug delivery over an extended period, enhancing the efficacy of genistein. This not only improves the therapeutic outcome but also offers a more comfortable treatment experience for patients, potentially increasing their compliance with the treatment regimen.

### 4.5 Solid Dispersion

Solid dispersion refers to a kind of solid dispersion formed by highly dispersing insoluble drugs in solid dispersion materials by certain methods (Huang and Dai 2014). Solid dispersion can highly disperse drugs to form molecular, colloidal, microcrystalline, or amorphous dispersion states, which can greatly improve the dissolution and absorption of drugs (Jin et al. 2012). Solid dispersion technology is a technology that the drug is highly dispersed in another solid carrier material by a certain method to form a solid dispersion. The encapsulation of genistein in solid dispersions can enhance its therapeutic efficacy by improving its dissolution profile and facilitating in vivo absorption.

It has been studied that the genistein‐nano‐silica solid dispersion was prepared by the solvent method and the conversion of genistein was (93.47 ± 2.40) % (Andhariya and Burgess 2016). Qiu et al. (2024) prepared solid dispersions of genistein using the solvent rotary evaporation method, with polyvinylpyrrolidone K30 serving as the carrier. The solid dispersion of genistein could represent a promising formulation strategy for enhancing the dissolution rate and improving oral bioavailability. Serebrenik et al. (2023) developed an amorphous solid dispersion of genistein using hot melt extrusion for prophylactic administration to improve the survival rate of samples after radiation exposure.

A new type of drug preparation, solid dispersion, was prepared by highly dispersing genistein in periodontal tissue engineering scaffolds. This formulation enhances the dissolution, sustained release, and targeted delivery of genistein, making it more effective in periodontal tissue regeneration engineering. Additionally, genistein's pharmacological activities, including its anti‐inflammatory properties and osteogenic differentiation capabilities, are likely to be more pronounced with this drug preparation. Therefore, this innovative solid dispersion approach has the potential to significantly improve the outcomes of periodontitis treatment and periodontal tissue regeneration.

### 4.6 Microsphere

Microspheres are drug‐loaded systems in which drugs are dispersed or adsorbed on carriers, and microspheres of 1–500 μm are formed by microencapsulation technology, and then various dosage forms are made according to the clinical route of administration and therapeutic use (Cai, Chen, et al. 2013). This special drug dosage form enables the drug to be effectively fixed and dispersed in the micro‐skeleton of polymer materials, thus possessing a series of unique properties and application advantages. As a new type of drug dosage form, microspheres show a wide range of application potential in the field of drug carriers. They have a variety of administration routes, including oral, injection, and topical administration, so as to meet the needs of different disease treatments.

In order to address the solubility issues of genistein and facilitate controlled release, studies have been conducted on the microencapsulation of genistein extracted from soybeans using spray‐drying techniques to obtain solid dispersions. This method has shown promise in overcoming the solubility limitations of genistein and enhancing its therapeutic potential (Panizzon et al. 2014). In addition, chitosan microspheres loaded with genistein were successfully fabricated utilizing the double emulsion method. This technique yields microspheres characterized by excellent shape uniformity, high production efficiency, and notable sustained release capabilities (Wu and Li 2002b).

**FIGURE 4:**
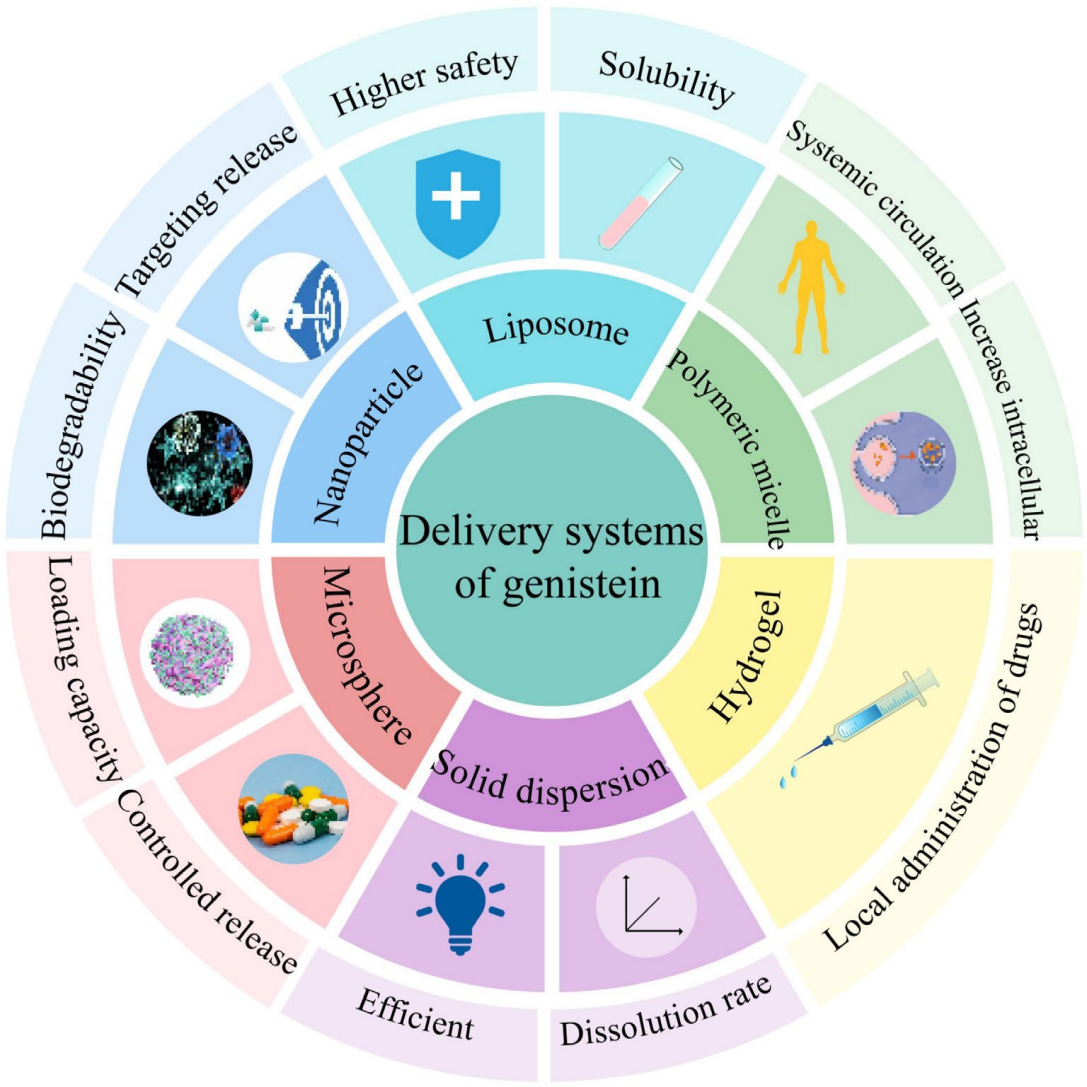
Partial drug delivery system of genistein and its advantages. To enhance genistein's bioavailability, various strategies have been employed, with nanoparticles, liposomes, polymer micelles, hydrogels, solid dispersions, and microspheres being the most common.

Microsphere‐based scaffolds are regarded as suitable materials for bone and cartilage injury repair due to their microporous structure, which offers ample internal space conducive to cell proliferation and other cellular activities. In the context of periodontal disease treatment, microsphere scaffolds have the potential to enhance the bioavailability of genistein. By incorporating genistein into these scaffolds, more effective delivery of the drug to the target site can be ensured, thereby augmenting its therapeutic efficacy. Consequently, microsphere scaffolds present a promising strategy for improving the treatment of periodontal disease through optimized genistein delivery. Figure 4 shows the partial drug delivery system of genistein and its advantages.

## 5 Discussion

Our research focuses on the investigation of the diverse pharmacological effects of genistein within the pathogenic mechanisms of periodontitis. Through rigorous analysis, it is possible to identify key molecular targets of genistein's action, which may offer novel perspectives for the prevention and treatment of periodontitis. Furthermore, recent studies have demonstrated that various drug delivery systems for genistein enhance its bioavailability, subsequently improving therapeutic outcomes. Consequently, genistein can be regarded as an efficacious therapeutic adjuvant.

The soft–hard tissue interface of the human periodontium plays a critical role in maintaining periodontal homeostasis and is indispensable for normal oral function. This interface is shaped by a complex interplay of physical, chemical, and biological factors. Periodontal ligament stem cells (PDLSCs) are localized at the interface between the soft and hard tissues of the periodontium (Dang et al. 2024). Additionally, estrogen stimulates PDLSCs and enhances their osteogenic differentiation, with these effects demonstrating a concentration‐dependent relationship (Cai, Yuan, et al. 2013). Notably, genistein may modulate estrogen‐mediated processes in the treatment of periodontitis, thereby contributing to the regulation of homeostasis at the interface between the soft and hard tissues of the human periodontium.

Dental calculus comprises inorganic components and an organic matrix derived from saliva, gingival crevicular fluid, and bacterial byproducts (Wei et al. 2024). Dental calculus serves as a local contributing factor in the pathogenesis of periodontitis (D'souza et al. 2023). The primary goal of periodontitis treatment is to eliminate inflammation, halt the progression of the disease, and ultimately facilitate the regeneration of tooth‐supporting tissues. Conventional basic scaling is effective in removing only a portion of dental calculus and plaque, indicating certain limitations in its efficacy. Genistein, a natural compound with diverse biological activities, is employed as an adjuvant therapeutic agent in the management of periodontitis. The pharmacological properties and mechanisms of genistein in the treatment of periodontal disease are primarily centered on two key aspects: inhibiting periodontal soft tissue destruction and inhibiting alveolar bone destruction.

Genistein not only inhibits periodontal soft tissue destruction but also has been shown to protect alveolar bone by inhibiting osteoclasts and promoting osteogenesis. It down‐regulates the expression of inflammatory mediators by regulating various signaling pathways such as NF‐κB, MAPK, and iNOS/NO and inhibits the damage of inflammation to periodontal tissue. In terms of protecting alveolar bone, genistein can replace estrogen to effectively prevent the reduction of alveolar bone height caused by estrogen deficiency in early menopause and reduce the risk of tooth loss. Genistein can not only act on the OPG/RANK/RANKL signal axis, reduce the RANKL/OPG ratio, and inhibit the activity of osteoclasts but it can also effectively reduce the bone loss of ovariectomized rats. It can also activate the MAPK signaling pathway in mouse bone marrow mesenchymal stem cells in a dose‐dependent manner, promote new bone formation, and play a role in bone protection.

The disruption of the balance between osteoblasts and osteoclasts by inflammatory cytokines constitutes the primary cause of bone loss (Usui et al. 2021). In the periodontal inflammatory microenvironment, bacterial lipopolysaccharide interacts with monocytes to produce a variety of cytokines. These cytokines (such as TNF‐α, IL‐6, IL‐1β, and IL‐17) play a role in the bone destruction pathway of periodontitis and can cause bone resorption by regulating osteoclast formation (Ramadan et al. 2020). In addition to inducing the inflammatory response, TNF‐α, IL‐6, IL‐1β, and IL‐17 can regulate osteoclast differentiation. TNF‐α can induce osteoclast differentiation of macrophages by directly increasing the expression of RANK in macrophages or enhancing RANKL signal transduction (Kitaura et al. 2013). IL‐6 not only promotes the differentiation of bone marrow‐derived macrophages into osteoclasts but also synergizes with IL‐1 to accelerate bone loss in ovariectomized rats (Dai et al. 2000). Moreover, the combination of TNF‐α, IL‐6, and IL‐1β significantly increased the ratio of RANKL/OPG in cells and enhanced osteoclast activity (Blaschke et al. 2018). The exact mechanism by which genistein inhibits ligation‐induced alveolar bone destruction is unclear. Various pro‐inflammatory mediators, such as NO and IL‐6, are present at a high level in the lesion site of periodontal disease and are associated with a higher severity of periodontal disease. In addition, both NO and IL‐6 possess the ability to activate osteoclasts and induce bone destruction (Matejka et al. 1998; Pelt et al. 2002). Therefore, regulating the excessive production of these destructive mediators seems to have a protective effect on alveolar bone destruction caused by periodontal disease.

The low water solubility of genistein limits its application in periodontitis. All kinds of new genistein drug delivery systems show excellent solubility, targeting, and sustained release properties, which can overcome the problems of poor water solubility of drugs. It can accurately deliver drugs to the lesion site, continuously and stably release drugs, and effectively prolong the action time of drugs. Given that genistein exhibits excellent pharmacological activities such as anti‐inflammatory and osteogenic promotion, its high melting point and low water solubility greatly limit its application potential in the treatment of periodontal disease. In order to solve this problem, we can explore the optimization of the drug delivery system of genistein in order to overcome its inherent physical obstacles and improve its solubility in saliva, which provides more choices for the product design of genistein in the field of periodontitis.

In a number of basic studies, genistein is usually administered immediately after animal periodontitis model establishment. However, in the clinical environment, genistein may be used in the development or middle and late stages of periodontal disease. Consequently, further research is warranted to thoroughly evaluate the potential of genistein as a therapeutic agent for patients suffering from periodontitis. Although genistein has shown multi‐dimensional therapeutic potential in periodontitis‐related research, there are few studies on its application in the treatment of periodontitis patients in clinical trials, and its development potential, related mechanism, and safety remain to be further studied.

## 6 Overall Conclusions and Future Perspectives

Genistein, a versatile phytoestrogen, exhibits a wide array of biological activities in mammals, functioning as both an estrogen agonist and antagonist. However, its health‐promoting attributes transcend its estrogenic mimicry. Preclinical studies have documented numerous pharmacological activities of genistein, encompassing anti‐inflammatory, antioxidant, antibacterial, anti‐osteoporotic, anti‐tumor, and estrogen‐like effects. In the context of periodontitis, genistein demonstrates a potent ability to suppress the mRNA and protein expression of inflammatory mediators, including IL‐1β, TNF‐α, COX‐2, IL‐6, IL‐17a, ICAM‐1, and PGE_2_, thereby exhibiting significant anti‐inflammatory activity in the periodontal inflammatory response. Furthermore, genistein modulates bone metabolism by inhibiting the expression of RANKL and augmenting the expression of OPG, while simultaneously enhancing the expression of AKP and BGP. These actions collectively contribute to bone protection and mitigate the progression of periodontal tissue destruction. Emerging evidence from existing research robustly supports the therapeutic efficacy of genistein in the prevention and treatment of periodontal disease, highlighting its diverse pharmacological activities in periodontal tissue. However, the current body of research examining the effects of genistein on periodontal disease remains limited, and the underlying mechanisms of its potential therapeutic action have yet to be fully elucidated. Further research is needed to provide a more scientific basis for clinical periodontal treatment.

Genistein possesses antibacterial properties; however, to date, there have been no reports on the relationship between genistein and the major pathogenic bacteria associated with periodontitis. The antibacterial capabilities of genistein present a promising avenue for further research into its potential applications in the treatment of periodontitis‐related pathogens. By examining the interaction between genistein and pathogenic bacteria associated with periodontitis, new insights may be gained into the mechanisms by which genistein inhibits bacterial growth and virulence, ultimately paving the way for the development of innovative therapeutic strategies for this condition.

The limited aqueous solubility of genistein hampers its application in the treatment of periodontitis, prompting a surge in research aimed at enhancing its bioavailability. A variety of novel drug delivery systems for genistein have been developed to tackle the challenge of its poor solubility. Local administration of these systems not only mitigates systemic side effects but also ensures targeted delivery of the drug to the site of the lesion, thereby maximizing its therapeutic efficacy. Moving forward, the conduct of additional clinical trials and studies utilizing genistein preparations with superior bioavailability holds the promise of unlocking new frontiers in the field of periodontal research.

## Author Contributions

**Yujun Lu:** conceptualization (equal), methodology (lead), visualization (lead), writing – original draft (lead), writing – review and editing (equal). **Yuye Liang:** methodology (equal), resources (equal), software (equal). **Zhiyuan Li:** funding acquisition (equal), investigation (equal), visualization (equal). **Wenxiu Li:** funding acquisition (equal), visualization (equal). **Changjun Huang:** data curation (equal), formal analysis (equal). **Yajing Wang:** conceptualization (equal), funding acquisition (lead), methodology (equal), supervision (equal), writing – review and editing (equal). **Guangcheng Wang:** conceptualization (equal), funding acquisition (equal), methodology (equal), supervision (equal), visualization (equal), writing – review and editing (equal).

## Conflicts of Interest

The authors declare no conflicts of interest.

## Data Availability

Even though adequate data have been given in the form of tables and figures, all authors declare that if more data are required, then the data will be provided on a request basis.
